# Giant cervicothoracic malignant peripheral nerve sheath tumor in neurofibromatosis type 1: a case report of multidisciplinary management and literature review

**DOI:** 10.3389/fonc.2026.1883580

**Published:** 2026-07-10

**Authors:** Jie Zheng, Wenbo Duan, Hongxun Sang, Xiaohai Fan, Kuangwen Li, Lei Cao

**Affiliations:** 1 Department of Orthopaedics, China-Japan Union Hospital of Jilin University, Changchun, Jilin, China; 2 Department of Orthopaedics, Shenzhen Hospital of Southern Medical University, Shenzhen, Guangdong, China

**Keywords:** en bloc resection, malignant peripheral nerve sheath tumor, multidisciplinary approach, neurofibromatosis, vascular reconstruction

## Abstract

**Introduction:**

Neurofibromatosis type 1 (NF1) is a genetic disorder predisposing patients to malignant peripheral nerve sheath tumors (MPNSTs), which represent a leading cause of mortality with poor therapeutic outcomes. Large MPNSTs with NF1 are extremely rare, and only a few clinical reports are published on this disease. We describe a case of giant cervicothoracic MPNST with major vessels encased and present the clinical profiles, radiological findings, operative management, and follow-up data, along with a literature review.

**Case report:**

A 30-year-old male patient complained of an incidentally discovered painful mass in the right neck that had been progressively enlarging, accompanied by right hand weakness and intrinsic muscle atrophy. Contrast-enhanced MRI revealed a giant tumor closely related with the right brachial plexus and intervertebral foramina of C6–T2. The right subclavian artery and internal jugular vein were tightly encased by the tumor. A multidisciplinary approach incorporating advanced 3D imaging, preoperative vertebral artery embolization, and en bloc resection with vascular reconstruction achieved successful tumor removal while preserving neurological function. The patient received adjuvant radiotherapy and was followed up on the 6th, 12th and 18th months postoperatively. There were no complaints or signs of recurrence.

**Conclusion:**

The favorable 18-month clinical and radiologic outcome provides a valuable reference for managing similar high-risk cases, though long-term surveillance remains crucial due to the high recurrence potential of MPNSTs. This report contributes to the limited literature on radical surgical management of NF1-associated MPNSTs and emphasizes the critical balance between oncological control and functional preservation in these challenging cases.

## 1 Introduction

Neurofibromatosis type 1 (NF1) is an autosomal dominant disorder caused by mutations in the “NF1” tumor suppressor gene located on chromosome 17q11.2, affecting approximately 1 in 2500 to 3500 individuals worldwide (1, 2). This disease is characterized by café-au-lait macules, cutaneous neurofibromas, and skeletal abnormalities, with up to 50% of cases arising *de novo* (3). A critical complication of NF1 is the malignant transformation of plexiform neurofibromas into malignant peripheral nerve sheath tumors (MPNSTs), occurring in 8-13% of patients and representing the leading cause of mortality in this NF1 population (4, 5). MPNSTs are highly aggressive sarcomas with a 5-year survival rate of 20-50% and are often resistant to conventional chemical therapies (6, 7). The diagnosis of MPNST remains challenging due to its histological heterogeneity and lack of specific biomarkers and therefore requires an integrated clinicopathologic analysis based on clinical context, imaging, morphology and immunohistochemistry outcomes (8). Surgical resection with negative margins remains the cornerstone of treatment, though achieving complete resection is often complicated by the close relation between the tumor and major blood vessels and critical nerves (9).

Here we report an apparently rare and clinically challenging scenario: a 30-year-old male with NF1 developed a gigantic cervicothoracic MPNST originating from a pre-existing neurofibroma, as confirmed by immunohistochemistry. This case is notable for its anatomical complexity, including the encasement of subclavian artery and vertebral artery, as well as a symptomatic presentation with progressive neurological deficits (4, 10). Such large, vascularized MPNSTs in critical locations pose significant diagnostic and therapeutic dilemmas, especially in distinguishing benign neurofibromas from malignant transformations and managing surgical risks like catastrophic hemorrhage and irreversible neural impairment (11).

## 2 Case presentation

### 2.1 Patient information

A 30-year-old male complained of a swelling mass in the right neck, accompanied by occasional self-resolving forearm numbness. The mass was initially egg-sized and progressively enlarged to the size of a small ball, accompanied by right hand weakness and intrinsic muscle atrophy. He felt a predominantly decreased endurance for physical activity. Due to the significant impact on the quality of life and inability to work, he came to the clinic for further treatment. He had been diagnosed as neurofibromatosis type 1 (NF1) for over 20 years and underwent posterior spinal deformity correction surgery 13 years ago. The patient’s grandfather was diagnosed with NF1, with typical scattered café-au-lait spots on the skin. His parents and other direct relatives had no history of neurofibromatosis and other genetic disorders.

### 2.2 Clinical findings

**Figure 1:**
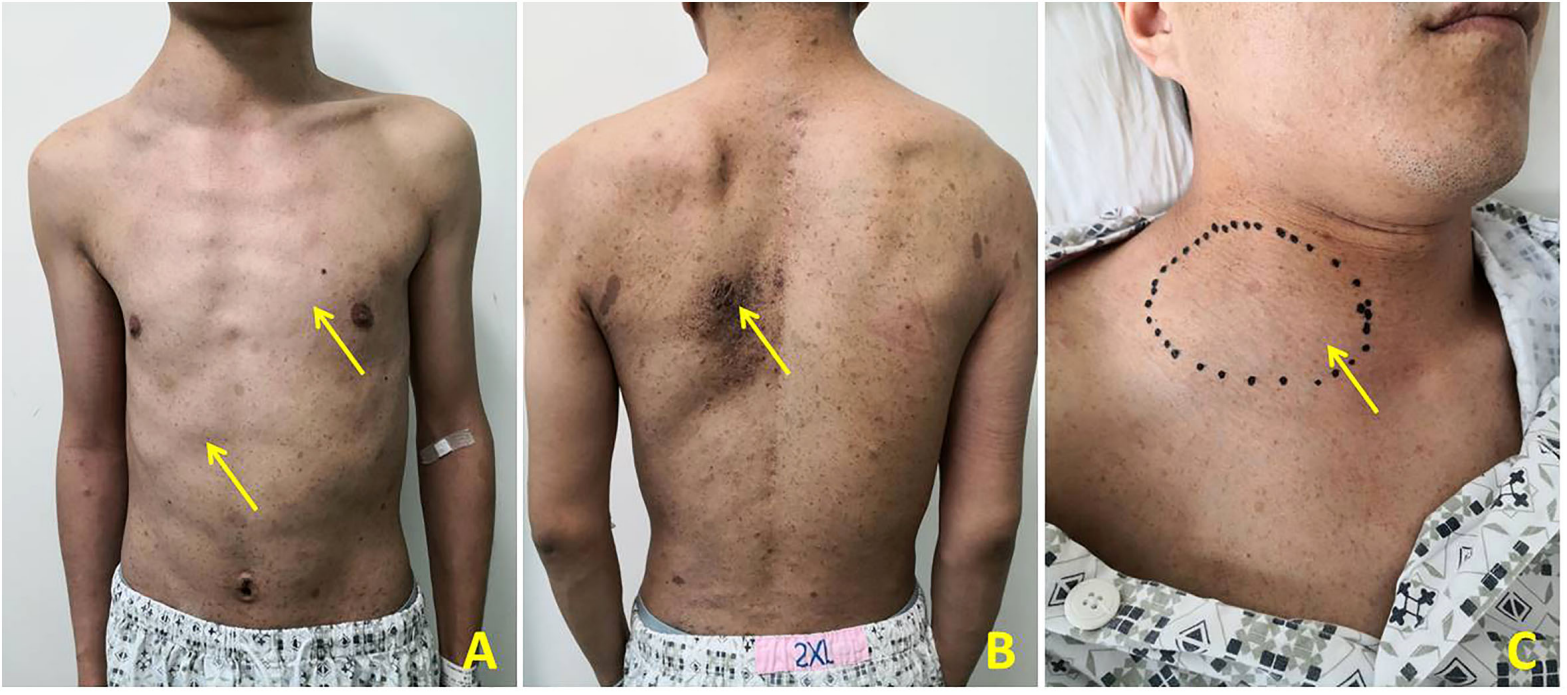
**(A)** Scattered café-au-lait spots on the skin. **(B)** An ulcerated plexiform neurofibroma was noted in the left scapular region. **(C)** A firm, roughly oval-shaped mass measuring approximately 15 cm × 10 cm in the right neck.

Scattered café-au-lait spots were present on the skin, and two ulcerated plexiform neurofibromas were noted in the left scapular region of the back and the left buttock (**Figures 1A, B**). Physical examination revealed a firm, roughly oval-shaped mass measuring approximately 15 cm × 10 cm in the right neck (**Figure 1C**). Local tenderness and percussion pain were present, while Tinel’s sign was negative. Moderate atrophy of the hand intrinsic muscles and muscle weakness (III/V) were observed.

### 2.3 Diagnostic assessment

**Figure 2:**
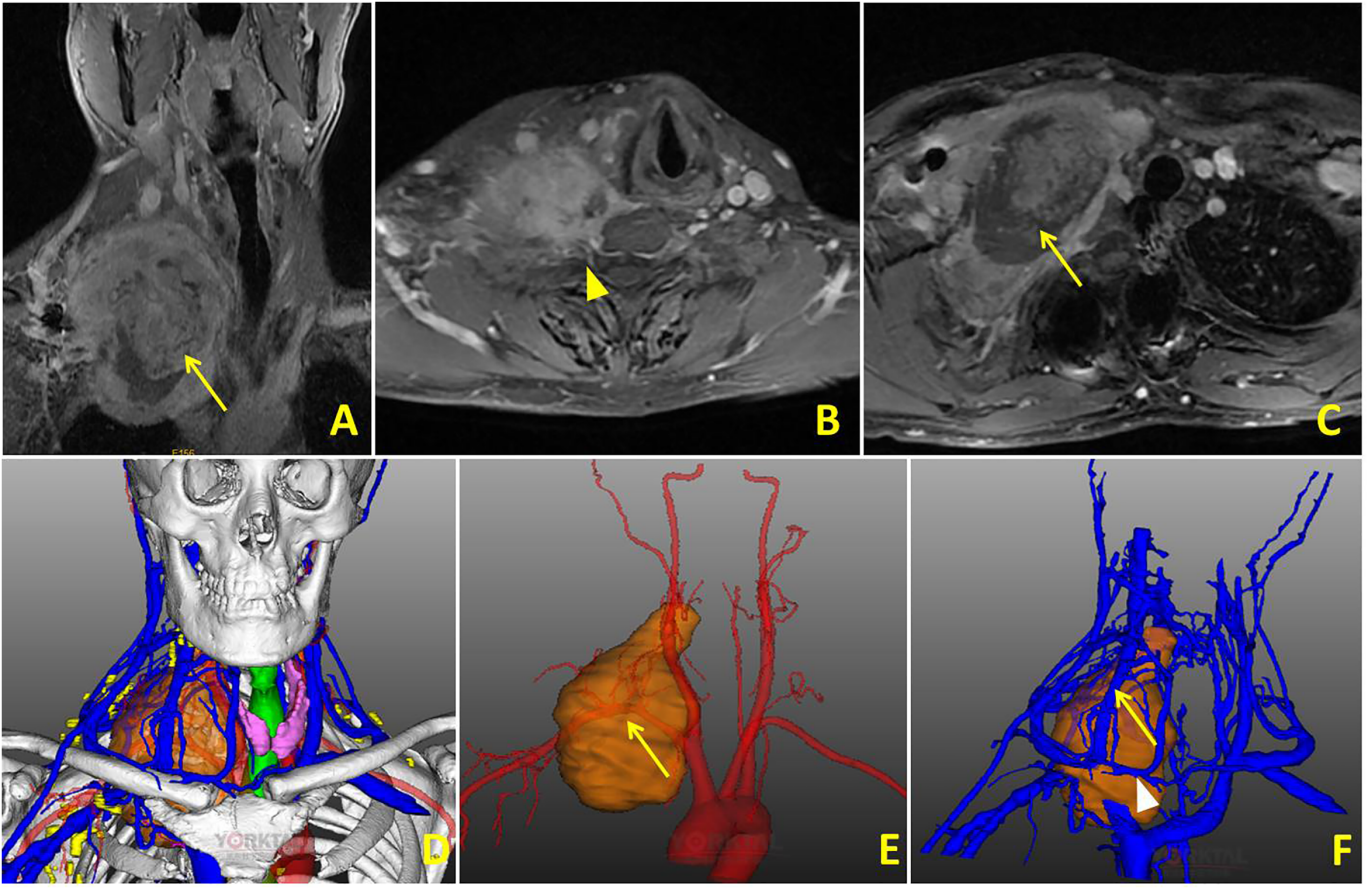
**(A)** Contrast-enhanced MR revealed a giant tumor extending from epiglottis level superiorly to the third intercostal space inferiorly (yellow arrow). **(B)** T2-weighted phase showed the tumor extending into the spinal canal through the intervertebral foramen (yellow triangle). **(C)** Areas of hemorrhage and necrosis within the tumor showed heterogeneous signals (yellow arrow). **(D)** A giant MPNST in the right thoracocervical region with severe adhesion to adjacent structures. **(E)** The clavicular segment of the right subclavian artery (yellow arrow) went through the tumor, with nearly normal physiological function. **(F)** The right internal jugular vein (yellow arrow) was pushed anteriorly, and the right subclavian vein (white triangle) had almost lost its physiological function, with collateral circulation established.

Contrast-enhanced MRI revealed a tumorous lesion in the medial space of the right sternocleidomastoid, measuring approximately 140 mm × 90 mm×90mm. It further demonstrated the tumor’s close relationship with the right brachial plexus and intervertebral foramina of C6–T2 (**Figures 2A–C**). Neck and chest contrast-enhanced CT showed the tumor was supplied by the right subclavian artery and right vertebral artery. The right common carotid artery, trachea, and other adjacent structures were compressed to the left. The right subclavian artery and internal jugular vein were tightly encased by the tumor. A three-dimensional model was reconstructed on the basis of CT data using Mimic V20.0 (Materialise, Belgium), facilitating visualization of tumor size, morphology, and surgical planning (**Figures 2D–F**; **Supplementary Videos 1**, **2**). Fluorodeoxyglucose-positron emission tomography –CT (^18^FDG PET-CT) demonstrated hypermetabolism of the lesion and showed no evidence of distant metastasis.

The needle biopsy strategy was reviewed by multidisciplinary team. The giant size and superficial palpability of the tumor facilitated a straightforward trajectory for needle biopsy. The ultrasound-guided biopsy targeted the most suspicious enhancing region (peripheral and transitional zone) identified on MRI. Microscopically, the tumor consisted of spindle cells organized into fascicular and interlacing patterns, accompanied by multifocal coagulative necrosis, hemorrhage, and mucoid degeneration. Immunohistochemistry results were as follows: PGP9.5 (+), S-100 (+), SOX10 (–), EMA (–), CK (–), Bcl-2 (+), CD99 (+), CD34 (–), CD117 (–), p-MEK (–), Desmin (–), Dog-1 (–), INI-1 (+), H3K27me3 (-), and Ki67 (70%+ in hotspots). Even with atypical results (SOX10 negative, S100 positive), various critical biomarkers with the loss of H3K27me3 expression helped exclude other important spindle-cell sarcomas such as synovial sarcoma (H3K27me3-retained, EMA, CK and INI-1 positive), leiomyosarcoma (typically Desmin and SMA positive), melanoma (typically SOX10 and S-100 positive), sarcomatoid carcinoma (EMA and CK positive), solitary fibrous tumor (typically CD34, Bcl-2 and CD99 positive, low Ki67) and gastrointestinal stromal tumor (DOG1 and CD117 positive). Combined with the clinical and radiographic outcomes, the pathological results indicated a spindle cell malignant peripheral nerve sheath tumor (MPNST).

### 2.4 Vertebral artery embolization

**Figure 3:**
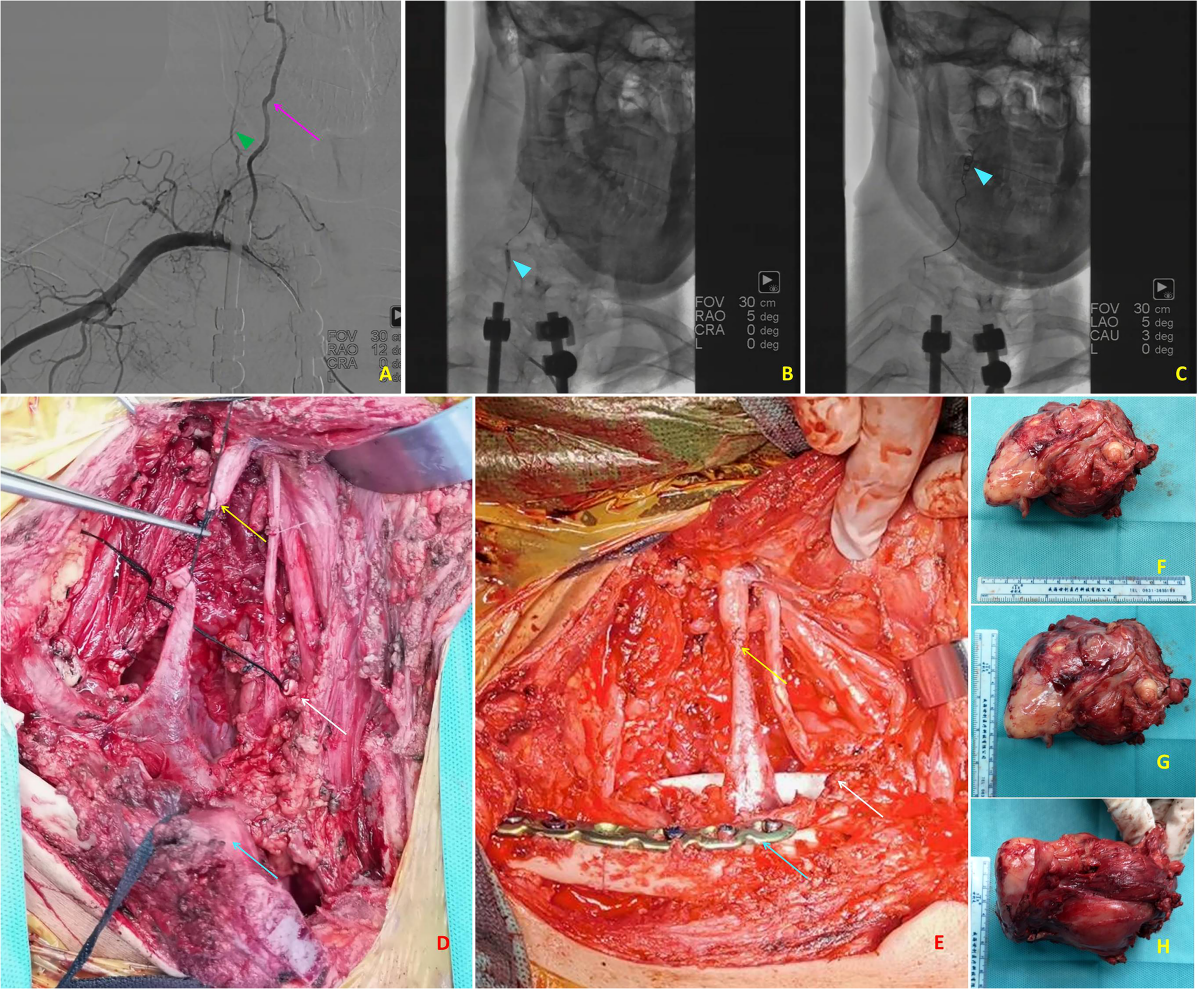
**(A)** Right vertebral artery (green triangle) and common carotid artery (pink arrow) were visualized by the intraoperative DSA. **(B)** Test occlusion was performed via a 3 mm balloon catheter (blue triangle). **(C)** Detachable coils (blue triangle) were deployed in the right vertebral artery. **(D)** A clavicle osteotomy was performed (blue arrow) and the tumor was excised together with part of the subclavian artery (white arrow) and internal jugular vein (yellow arrow); **(E)** Artificial vascular reconstruction of subclavian artery (white arrow), clavicle reconstruction with titanium internal fixation (blue arrow), and end-to-end anastomosis of internal jugular vein (yellow arrow); **(F-H)** Complete excision of the tumor, measuring approximately 140mm × 90mm × 90mm.

Notably, contrast-enhanced CT revealed a dominant left vertebral artery with tumor feeders from the right vertebral artery and thyrocervical trunk. To minimize intraoperative hemorrhage, the MDT decided on right vertebral artery embolization. An experienced vascular surgeon performed the embolization under neuro-monitoring. During the maneuver, a modified Seldinger technique (12, 13) was used to puncture the right femoral artery, followed by the insertion of a 5F short arterial sheath. After heparinization, a 5F pigtail catheter was advanced to perform aortic arch angiography. Intraoperative digital subtraction angiography (DSA) revealed that the patient had a type I aortic arch. The left vertebral artery, originating from the left subclavian artery, was the dominant artery. The right vertebral artery was visualized but was apparently smaller (**Figure 3A**). A 3 mm balloon catheter was advanced over a micro-guidewire into the right vertebral artery for a 10-minute test occlusion (**Figure 3B**). No signs of intracranial ischemia were observed, thereby rendering the right vertebral artery suitable for embolization. A 2.4F microcatheter was then navigated into the right vertebral artery to the level of the C3 vertebra, through which detachable coils (6×200mm Interlock, Boston Scientific Corporation) were deployed (**Figure 3C**). Post-embolization angiography confirmed successful occlusion. The patient reported no headache or neural dysfunction after embolization.

### 2.5 Surgical resection

On the next day, surgical tumor resection was performed through a combined approach via partial sternotomy and mid-clavicle osteotomy. The tumor was measured approximately 140 mm × 90 mm and extended from the epiglottis level superiorly to the third intercostal space inferiorly, with approximately half of its volume intruding into the thoracic cavity. The right subclavian artery, the origin of vertebral artery and phrenic nerve were tightly encased by the tumor and could not be stripped safely. The brachial plexus was compressed posteromedially with severe adhesions to the tumor. Thus the tumor resection margins were defined by the following anatomical structures: the anterior margin was the clavicle, the shifted internal jugular vein, and the subclavian vein; the posterior margin was the compressed common carotid artery and the roots of the brachial plexus (C6-T1); the medial margin was the brachiocephalic trunk and the vagus nerve; the inferior margin corresponded to the third intercostal space; and the lateral margin was the shifted brachiocephalic veins and scalene muscles. The brachial plexus cords were carefully dissected and freed proximally to the root level. After complete dissociation and protection of the peripheral structures (brachial plexus, common carotid artery, vagus nerve, and subclavian vein) (**Figures 3D, E**), the encased internal jugular vein, right phrenic nerve, clavicular segment of the subclavian artery, and origin of the vertebral artery were ligated and resected en bloc with the tumor. A 70 mm × 8 mm Gore-Tex graft (W.L. Gore & Associates, USA) was used for subclavian artery reconstruction via a running suture technique with 6–0 Prolene suture, and the remaining internal jugular vein was anastomosed end-to-end directly. The reconstructed subclavian artery was well perfused with strong pulsation, as confirmed by operative surgeons. The clavicle and sternum were repositioned and fixed with locking plates and nails (**Figure 3E**). After irrigation and careful hemostasis, a chest tube was placed in the 6th intercostal space at the mid-axillary line. The cervicothoracic incision was closed layer by layer. The whole operation procedure had been recorded and uploaded as **Supplementary Video 3**. The estimated intraoperative blood loss was approximately 2500 ml, with 5550ml of fluid supplement, 800 ml of plasma, 10 units of red blood cells, and 6 units of cryoprecipitate. Postoperative examination confirmed intact sensation and motor function in all limbs.

**Figure 4:**
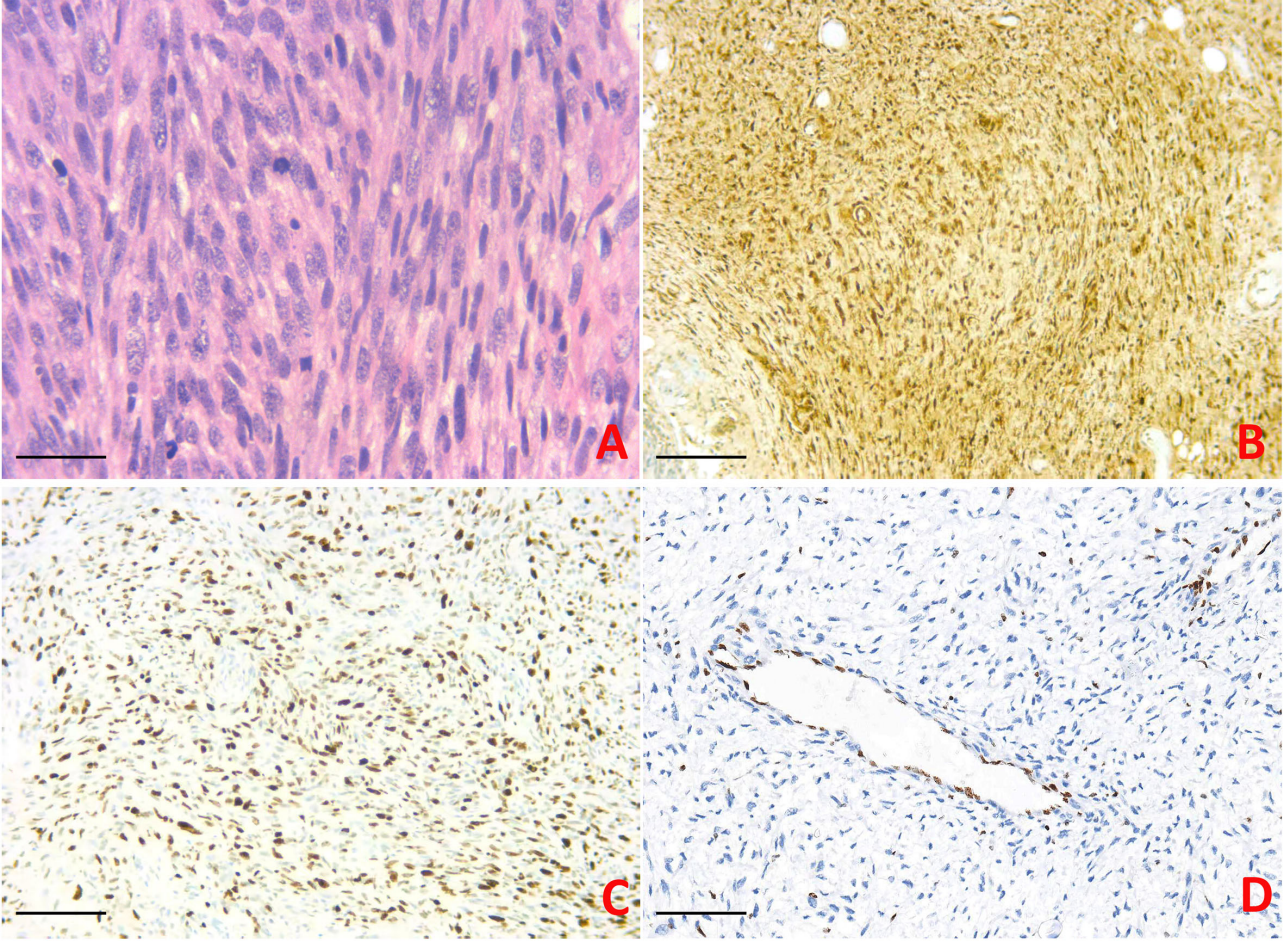
**(A)** H & E stained slides showed spindle cells arranged in fascicular and interlacing patterns with hyperchromatic, pleomorphic nuclei with hypercellularity (400×, Scale bar = 25μm). **(B)** Immunohistochemical staining showed positive staining with S-100 protein (20×, Scale bar = 100μm). **(C)** Ki-67 value was 70%+ in hotspots, demonstrating high tumor proliferation (20×, Scale bar = 100μm). **(D)** Immunohistochemistry for H3K27me3 demonstrates loss of nuclear staining in tumor cells, while endothelial cells are retained as internal positive controls (20×, Scale bar = 100μm).

The tumor, along with the intact capsule, was resected without rupture (**Figures 3F–H**). Postoperative pathological findings indicated that the tumor was FNCLCC grade 3 (mitotic count: >20/10 HPF, necrosis: 30%) (14), without evidence of lymphovascular invasion. With a perineural origin, the tumor was confirmed as malignant transformation of a neurofibroma into an MPNST. Margin status was microscopically negative (R0 resection), with a closest margin distance of 5 mm at the interface. The immunohistochemistry results were consistent with preoperative findings (**Figures 4A–D**).

### 2.6 Adjuvant radiotherapy

After surgery, we conducted a subsequent multidisciplinary consultation, and the consensus recommendations were as follows: based on the complete resection of the tumor with negative margins and the absence of metastatic lesions, postoperative local radiotherapy was indicated. However, due to the tumor size and high Ki67 value (70%+ in hotspots), we still recommended a chemotherapy regimen of doxorubicin combined with ifosfamide after radiotherapy. As the c-Kit and MEK were negative, conventional targeted therapies were not recommended. The patient vigorously chose to receive local radiotherapy alone considering his own nutritional status and the toxic side effects of chemotherapy agents.

Radiotherapy was administered in 35 fractions to a total dose of 66 Gy (2 Gy per fraction) using intensity-modulated radiotherapy (IMRT), targeting the surgical bed with 2-cm margins. Organs at risk included the spinal cord (maximum dose 45 Gy), the brachial plexus, and the reconstructed vascular graft (dose limited to <50 Gy). Radiotherapy commenced 5 weeks post-surgery. No toxicity like dermatitis or dysphagia was observed.

### 2.7 Follow-up and outcomes

After surgery, no signs of pyrexia, hypotension, electrolyte imbalances, or other ischemia-reperfusion injury-related complications were observed. The patient exhibited strong radial artery pulses and no sensory deficits in the right upper limb, with motor function unchanged compared to preoperative function. Mild Horner’s syndrome (ptosis, facial anhidrosis, and enophthalmos) was noted, probably due to intraoperative manipulation of the cervical sympathetic trunk. As the unilateral phrenic nerve was resected, the patient developed a moderate right hemidiaphragm elevation on chest radiography, with mild dyspnea on exertion but no need for supplemental oxygen or pneumonia. Pulmonary function tests revealed a mild to moderate restrictive pattern (FEV1: 77.3% predicted FVC: 75.9% predicted). He was initiated on postoperative antiplatelet therapy with aspirin 100 mg daily. Enhanced-MRI confirmed complete tumor resection, functional artificial subclavian artery, and intact brachial plexus. 35 sessions of adjuvant radiotherapy was administered after discharge without systemic chemotherapy.

The long-term surveillance schedule was as follows: the clinical examination and MRI of the cervicothoracic region every 6 months for the first 2 years, and annually thereafter. Neck, chest and abdomen CT scans were performed every 6 months for the first 2 years, then annually. Doppler ultrasound of the reconstructed subclavian artery was performed annually. At the 6-month follow-up, intrinsic hand muscle strength had improved to grade V, with resolution of facial symptoms and no evidence of tumor recurrence on imaging. At the 12-month follow-up, the pulmonary function recovered significantly (FEV1: 90.3% predicted FVC: 88.5% predicted), enabling the patient to return to daily life and normal workload. The patient’s episode of care is listed in **Table 1** according to CARE guideline. However, given the limited follow-up period, long-term outcomes remain uncertain. Ongoing follow-up will further evaluate potential recurrence or progress.

**Table 1:** CARE timeline of patient’s episode of care.

Time	Event	Key Details	Motor Function	Pulmonary Function
November 2024	Symptoms onset	Patient developed a swelling mass in the right neck, accompanied by occasional self-resolving forearm numbness		
November 2024	Gradual aggravation	The mass progressively enlarged, accompanied by right hand weakness and intrinsic muscle atrophy		
November 11th, 2024	Initial presentation (local hospital)	MRI revealed a large cervicothoracic mass, suspecting a malignant tumor.		
December 15th, 2024	Referred to our hospital (tertiary center), preoperative workup	Contrast-enhanced MRI, contrast-enhanced CT and ultrasound-guided needle biopsy; pathology confirmed MPNST	Left intrinsic muscles: III/V	FEV1: 92.2% predicted FVC: 91.7% predicted
December 24th, 2024	Multidisciplinary team (MDT) discussion	Orthopaedics, thoracic surgery, neurosurgery, vascular surgery, anesthesiology, radiology, oncology jointly formulated the plan.	Left intrinsic muscles: III/V	FEV1: 92.2% predicted FVC: 91.7% predicted
December 29th, 2024	Vascular intervention	The patient received right subclavian artery angiography and vertebral artery embolization.	Left intrinsic muscles: III/V	FEV1: 92.2% predicted FVC: 91.7% predicted
December 30th, 2024	Surgical resection	Radical tumor resection achieved (R0). Mild Horner’s syndrome was noticed.	Left intrinsic muscles: III/V	FEV1: 77.3% predicted FVC: 75.9% predicted
February 3rd – March 21st , 2025	Adjuvant radiotherapy	The patient received adjuvant radiotherapy (35 sessions) without major complications.	Left intrinsic muscles: IV-/V	FEV1: 80.3% predicted FVC: 78.2% predicted
July 2025	6-month follow-up	MRI showed no local recurrence. Neural dysfunction completely resolved.	Left intrinsic muscles: V/V	FEV1: 84.6% predicted FVC: 85.1% predicted
December 2025	12-month follow-up	No local recurrence or distant metastasis. Patient was in healthy condition and returned to normal life.	Left intrinsic muscles: V/V	FEV1: 90.3% predicted FVC: 88.5% predicted
June 2026	18-month follow-up	No local recurrence or distant metastasis (**Supplementary Figure 1**). Patient was in healthy condition	Left intrinsic muscles: V/V	FEV1: 90.5% predicted FVC: 88.9% predicted

### 2.8 Patient’s perspective

Before admission, the patient complained of severe persistent painful swelling in the right neck region, accompanied by decreased endurance for physical activity. He felt fatigued after walking approximately 200 meters. Due to the significant impact on the quality of life and inability to work, he was admitted to our department for further treatment. After a definitive diagnosis, the patient strongly requested surgery to improve his quality of life. Postoperatively, there was no significant damage to neurovascular function, which gave the patient the confidence and motivation to initiate functional rehabilitation training. At the 12-month follow-up, the patient had fully recovered physical activity capacity and returned to work. He expressed satisfaction with the entire treatment process.

## 3 Discussion

### 3.1 Narrative review

Giant cervicothoracic MPNSTs with major blood vessels involved are extremely rare and pose enormous challenges to en bloc surgical resection. A narrative literature review was conducted to synthesize relevant clinical evidence on MPNSTs. A targeted search of databases (PubMed, MEDLINE, Scopus) was performed for articles published between January 2000 and May 2026. The search employed MeSH terms including “malignant peripheral nerve sheath tumor” and “MPNST”. Inclusion criteria were restricted to case reports or case series with histologically confirmed MPNST. Exclusion criteria included non-English articles, studies other than case reports (reviews, cohort studies, experimental research, etc.), abstracts without full text, and studies focused on other tumor types. A total of 3262 records were initially identified and 146 full-text articles were retrieved and assessed for eligibility. A PRISMA-style workflow diagram is available in **Supplementary Data Sheet 1.PDF**. We further made a comparison table (**Table 2**) summarizing previously reported cervicothoracic MPNSTs.

**Table 2:** Summary of studies describing cervicothoracic MPNSTs.

Author	Sex		Age	Location		Tumor size	FNCLCC grade		Blood vessels involvement	Neural involvement		Surgical procedure	Margin status
Navarro O, et al. (15)	F		19m/6m	Case 1: Left cervicothoracic mass Case 2: Retroperitoneal mass		Case 1: Not specified Case 2: Not specified	Case 1: Not specified Case 2: Not specified		Case 1: Left common carotid and subclavian artery compressed, vertebral artery occlusion Case 2: major celiac vessels encasement	Case 1: Left brachial plexus and phrenic nerve compressed. Case 2: Not specified		Case 1: Complete surgical resection and lung biopsy Case 2: Surgical resection of peritoneal mass	Case 1: R0 Case 2: R0
Imazu M, et al. (16)	F		12y	Right supraclavicular region		8×8×4 cm	G1		Not specified	Right vagal and cervical sympathetic nerve compressed		Surgical resection with vagal and sympathetic nerve resected extensively	R2
Dartnell J, et al. (17)	F		28y	Left supraclavicular mass invading the thoracic inlet		13.5×9×6 cm (371g)	Not specified		Left subclavian artery compressed	Lower cord of brachial plexus encased		En bloc resection with lower cord of brachial plexus, via a median sternotomy with supraclavicular extension and rib excision	R0
Harrison W, et al. (18)	M		54y	cervicothoracic and paraspinal dumbbell tumor (C7-T5)		11.3×7×7 cm	Not specified		right common carotid artery and subclavian vasculature	Vagus nerve encased;		Complete surgical resection with clavicle resection and posterolateral thoracotomy	R1
Yan S, et al. (19)	M		27y	Right thoracic inlet (NF and right clavicle (MPNST)		Thoracic inlet mass: 6.4×4.7 cm; Clavicular mass: 2.3×2 cm	Not specified		Right brachiocephalic vein, common carotid artery, jugular vein, and subclavian vessels compressed	Not mentioned		Thoracoscopy-assisted tumor resection with right clavicle and the 1st rib partially resected	R0
Samanci Y, et al. (20)	M		29y	Extended from left C7-T1 neural foramen into costoclavicular area		6×3×3 cm	G2		Not specified	Left brachial plexus compressed		C7-T1 laminectomy; Gross-total resection of intraspinal part, near-total excision of paraspinal components	R2
Our case	M		30y	Right cervicothoracic mass extended from C4-T4 level		14×9×9 cm (4500g)	G3		Right subclavian artery and internal jugular vein encased, common carotid artery, trachea and subclavian vein compressed	Right phrenic nerve encased, brachial plexus compressed		Vertebral artery embolization. En bloc resection with subclavian artery and phrenic nerve, via a median sternotomy and mid-clavicle osteotomy	R0
Author		Reconstruction			Adjuvant therapy			Recurrence and metastasis			Outcomes		
Navarro O, et al. (15)		Not mentioned			Case 1: Chemotherapy Case 2: Chemotherapy and radiotherapy			Case 1: Progressive metastasis Case 2: Recurrence in pancreas			Case 1:Died after 22 months Case 2: Died 3 months after surgery		
Imazu M, et al. (16)		Not mentioned			No adjuvant therapy			No recurrence or metastasis at 12-month follow-up			Hoarseness and Horner’s syndrome. Survived at 12-month follow-up		
Dartnell J, et al. (17)		Not mentioned			Adjuvant radiotherapy			Multiple pleural and pulmonary metastases			Persistent weakness and sensory loss in C8/T1. Died 3 months postoperatively		
Harrison W, et al. (18)		Not mentioned			Adjuvant radiotherapy			No recurrence or metastasis			Syncopal episodes, right vocal fold paralysis. Survived at latest follow-up		
Yan S, et al. (19)		Not mentioned			Adjuvant chemoradiotherapy			No metastasis or recurrence at 3-month follow-up			Transient facial hypohidrosis. No nerve compression at 3-month follow-up		
Samanci Y, et al. (20)		Not mentioned			Adjuvant chemoradiotherapy			No recurrence or metastasis			Apparent neurological improvement at latest follow-up.		
Our case		Subclavian artery reconstruction and clavicle fixation			Adjuvant radiotherapy			No recurrence or metastasis			Mild Horner’s syndrome and weakness. Completely resolved at 6-month and 18-month follow-up.		

### 3.2 Epidemiological characteristics

MPNSTs are particularly significant in the context of NF1, as they represent a considerable proportion of soft tissue sarcomas in this population (21). Approximately 50% of MPNST cases are associated with preexisting NF1, highlighting the genetic predisposition that NF1 patients face. Meanwhile, the lifetime risk of developing MPNSTs in NF1 patients is estimated to be as high as 10%, compared to an incidence of only 0.001% in the general population (22). Notably, the risk of malignant transformation from plexiform neurofibromas to MPNSTs is estimated to be between 10-15%, emphasizing the critical need for vigilant monitoring in this NF1 population (23). The incidence of MPNSTs is multifactorial. Age, tumor size, and genetic predispositions have been identified as pivotal factors influencing the incidence of MPNSTs (24–26). The anatomical location of MPNSTs also influences the incidence and prognosis. MPNSTs frequently occur in the trunk and extremities, with a notable prevalence in areas where large nerve roots converge (22). As malignant sarcomas with complicated predisposing factors, MPNSTs are notorious for their aggressive nature, characterized by high recurrence and metastasis rates. Previous studies indicate that the 5-year survival rate for MPNSTs patients was rarely less than 50% (27).

### 3.3 Pathology and biomarkers

As a nerve-origin solid tumor, MPNST is typically white and fleshy and may show myxoid changes. Microscopically, it exhibits various morphologies and distinct subtypes, characterized by elongated, curved nuclei and scant cytoplasm, with common necrosis, mitosis, and hemorrhage (28). Even non-specific, the diffuse cellularity and conspicuous mitoses may ensure the differentiation of MPNST from atypical neurofibroma and nonneurogenic tumors (29).

MPNST lacks distinctive molecular markers and relies on immunohistochemistry for diagnosis. As one of the conventional biomarkers, S-100 negativity may indicate Schwann cell de-differentiation and predict malignancy, so its absence correlates with increased metastasis and mortality risk (30). SOX-10 has similar sensitivity as S-100 in most neural crest-derived tumors, but is poorly sensitive due to its variable expressions in MPNSTs (31). Ki67 levels above 10% may indicate MPNST (32), while CD34-positive cells decrease significantly in high-grade MPNSTs (33, 34). Nestin is a more sensitive marker for MPNST but cannot easily differentiate MPNST from desmoplastic melanoma either (35).

In recent years, several new MPNST markers have been identified in the molecular investigations of MPNST mechanisms. The complete loss of the CDKN2A-encoded cell cycle regulator p16 is a common finding in MPNST (32). This finding is consistent with Cui’s new study, which highlighted the need for genomic profiling for accurate MPNST diagnosis. Their work confirmed the RTK/RAS pathway alterations and identified a high rate (70.6%) of actionable mutations including CDKN2A, NF1 and FGFR1, thereby highlighting the potential for targeted therapies (36). p53 protein often accumulates in tumors due to its unique deregulation, and TP53 mutation is linked to high tumor grade in NF1-associated or sporadic MPNST (37), but they can also mislead diagnoses possibly (38). With the function of inhibiting proliferation and promoting apoptosis, p27 staining can be found in 33% of high-grade MPNST (39). However, p53, p16 and p27 lack systematic validation as reliable and specific biomarkers. One of the latest MPNST markers is H3K27me3, a downstream target of PRC2 mutation. H3K27me3 loss occurs in 30–90% of MPNST cases, more frequently in sporadic and radiation-associated types than in NF1-associated ones, and may indicate lower survival chances (40, 41). However, H3K27me3 loss does not differentiate MPNST from similar tumors like synovial sarcoma or fibrosarcomatous dermatofibrosarcoma protuberans (40).

Along with the recent advances, our understanding of MPNST pathobiology is shifting from a purely genetic model to an integration of significant epigenetic and genomic analyses. A novel study stratified MPNSTs into two actionable subgroups: MPNST-G1, characterized by SHH pathway activation and PRC2 deficiency, which may be amenable to treatment with SMO inhibitors (e.g., vismodegib, sonidegib); and MPNST-G2, driven by Wnt/β-catenin pathway activation associated with Wnt10A and RAC2 overexpression, and APC underexpression, suggesting vorapaxar as a potential therapeutic candidate (42). Recent studies showed that DNA profiling and methylation profiling can be analyzed to distinguish MPNST from benign tumors and facilitate MPNST classification with the help of methylome-based unsupervised clustering (43, 44). An innovative classification system was proposed including G1 (epigenetically driven, aggressive) and G2 (APC-driven) subtypes, suggesting that future therapeutic stratification may depend on the tumor’s methylation and chromatin remodeling status (44). Furthermore, the identification of non-canonical SHH signaling and specific miRNAs (miR-135b and miR-889) as key drivers of invasion provides actionable targets.

### 3.4 Clinical signs and symptoms

The clinical presentation of these tumors often includes rapidly enlarging masses, which may be accompanied by pain and neurological deficits (22). In some cases, the tumor’s location and size can lead to more severe complications, such as Horner’s syndrome, which arises from sympathetic trunk involvement, or Pancoast syndrome, characterized by shoulder pain and neurological deficits due to brachial plexus compression (45, 46). In addition, some patients may exhibit atypical symptoms that can complicate diagnosis. For instance, the presence of scoliosis or other musculoskeletal deformities can be associated with NF1 (47, 48) and may mask the underlying presence of a malignancy.

### 3.5 Radiographic signs

MRI is particularly advantageous due to its superior ability to demonstrate neural tissues, allowing for detailed visualization of tumor characteristics (49). MPNSTs often present as fusiform or lobulated masses that follow the course of major nerve trunks. A notable feature is the “tail sign,” where the tumor appears to connect to the nerve at both ends. On T1-weighted images (T1WI), MPNSTs typically show isointense or slightly hypointense signals. Areas of hemorrhage or necrosis within the tumor may show heterogeneous high or low signals, complicating the imaging interpretation (50, 51). Conversely, T2-weighted images (T2WI) usually reveal heterogeneous hyperintensity due to the coexistence of various tumor components, including cellular areas, myxoid stroma, necrotic tissue, and hemorrhage (52). The disappearance of the “target sign,” characterized by a central low signal surrounded by a high signal rim in benign neurofibromas, is a significant indicator of malignancy (53). Emerging imaging technologies, such as positron emission tomography (PET) combined with MRI (PET-MRI) and radiogenomics, have shown promise in enhancing the accuracy of distinguishing between benign and malignant tumors, thus facilitating earlier intervention and better patient outcomes (49).

### 3.6 Surgical treatments

The concept “en bloc resection” is a preferred strategy for the MPNST especially in extremities. However, for the malignant lesions that infiltrate vital structure within or adjacent to the mediastinum, no uniform consensus has been achieved (45, 54–57). The pathophysiological basis for such surgical challenges lies in the unique tumor microenvironment of NF1-associated lesions. Plexiform neurofibromas and their malignant counterparts are characterized by aberrant vasculature, including arteriovenous malformations and shunts, which significantly elevate hemorrhage risk (58). Furthermore, the underlying molecular pathogenesis of MPNSTs contributes to their aggressive behavior and chemotherapy resistance (59–61). In our case, the cervicothoracic location and massive size (140×90×90 mm, 4500 g) of this lesion represent an exceptionally rare presentation. The rapid growth and neurological deficits observed in this patient align with the classic alarm for malignant transformation NF1 (62, 63), and the degree of anatomical distortion and critical vascular encasement exceeded most reported cases (15, 17–19). Therefore, given the tumor’s malignant pathology and the need for clear surgical margins, an aggressive en bloc resection with vascular reconstruction was likely beneficial after MDT evaluation.

Various surgical approaches have been developed to achieve radical tumor resection while maximizing preservation of neurovascular function (64–68). The cervical supraclavicular approach is a traditional surgical technique for tumors located at the cervical root region and the upper thoracic inlet. This approach offers excellent exposure of critical neurovascular structures, such as the brachial plexus and subclavian vessels (64, 65). The cervicothoracic combined approach is performed via a cervical incision combined with partial sternotomy or posterolateral thoracotomy for dual exposure and en bloc removal (69). For tumors that predominantly invade the subclavian vessels or brachial plexus, a transmanubrial transclavicular approach is utilized involving resection of the medial clavicle and a portion of the manubrium, thereby facilitating extensive exposure of the brachial plexus and major vessels (70).

Given the extraordinary extension (C4-T4 level) of the MPNST in our case with subclavian vessels and brachial plexus tightly infiltrated, we performed extensive sternotomy and clavicle osteotomy to achieve sufficient exposure, thereby enabling meticulous dissection and minimizing the risk of iatrogenic injury. This aggressive yet precise strategy was guided by preoperative 3D modeling, which provided a comprehensive understanding of the tumor’s relationship with critical neurovascular structures (71). A multidisciplinary cooperation was indispensable for navigating these challenges and achieving a favorable oncological and functional outcome. Notably, the decision for preoperative selective vertebral artery embolization, rather than targeting the primary tumor feeders, was an innovation to mitigate the risk of catastrophic intraoperative hemorrhage and sudden cerebral ischemia, which is scarcely reported in prior reports (72). This novel strategy, guided by the tumor’s unique vascular anatomy and the presence of functional collateral circulation, exemplifies a less commonly employed application of embolization. The subclavian artery reconstruction and jugular vein anastomosis successfully preserved limb perfusion without sacrificing major vessels. These proactive strategies distinguish this case from previous reports of vascular complications in NF1 (73, 74).

### 3.7 Chemotherapy and radiotherapy

Chemotherapy is an option for unresectable or metastatic MPNST, with minimal response to adriamycin and ifosfamide (71). Doxorubicin is a first-line treatment, and its combination with ifosfamide shows superior clinical efficacy (75). Yet adjuvant chemotherapy has limited impact on survival or recurrence (76), although epirubicin and ifosfamide may extend median survival from 45 to 75 months after local therapies (77). Thus, despite the widespread use of adjuvant chemotherapy, its efficacy remains controversial and individualized. As is described in section 2.6, even though the MDT recommended a chemotherapy regimen, the patient vigorously declined systemic chemotherapy considering his own nutritional status and the potential toxic side effects of chemotherapy agents. This choice may influence the prognosis in the long-term follow-up.

Radiation therapy is recommended for high-grade lesions or lesion larger than 5cm (78) and provides excellent local control (79). Yet adjuvant radiation does not improve survival, despite some studies suggesting it can reduce tumor size for resection (80). Brachytherapy and intraoperative electron radiation therapy have also been utilized in MPNST therapy conventionally. Brachytherapy shows better local control than external beams and combining both may enhance results (81, 82).

## 4 Limitations

Firstly, this report is inherently limited by the relatively short follow-up duration of 18 months. Recurrence and metastasis may occur later even after apparently complete resection and negative surgical margin (R0 resection). We emphasize the need for long-term surveillance and acknowledge that our findings are preliminary.

Secondly, this highly specialized surgical strategy with multidisciplinary cooperation may not be generalizable to all centers. Future studies with larger cohorts and extended follow-up are needed to refine management guidelines. Nevertheless, this case underscores the critical importance of lifelong surveillance in NF1 patients and highlights the potential of integrated advanced techniques in managing these formidable tumors.

## 5 Conclusion

In summary, this case demonstrates the successful management of a rare, giant cervicothoracic MPNST in an NF1 patient through a meticulously planned, aggressive surgical strategy. It reinforces the necessity of a proactive, multidisciplinary approach for large, symptomatic MPNSTs, advocating for en bloc resection with vascular reconstruction to optimize oncological control and mitigate surgical risks. The strategic application of preoperative 3D modeling and selective embolization proved invaluable for planning and execution.

## Data Availability

The original contributions presented in the study are included in the article/**Supplementary Material**. Further inquiries can be directed to the corresponding author.

## References

[1] Mansilla-Polo M . Neurofibromatosis type 1. Med Clin (Barc). (2025) 164:107–8. doi: 10.1016/j.medcli.2024.04.012 38937219

[2] Mançano AD . Neurofibromatosis type 1. Radiol Bras. (2022) 55:VII–VIII. doi: 10.2139/ssrn.4752924 PMC8864685 35210668

[3] Moodley M Lopez KR . Neurofibromatosis type 1 - an update. Semin Pediatr Neurol. (2024) 52:101172. doi: 10.1016/j.spen.2024.101172 39622609

[4] Hartung TI Karimi Q Kluwe L Farschtschi SC . A case of neurofibromatosis type 1 with neurofibromatosis type 1-related and neurofibromatosis type 1-unrelated tumors: a case report. J Med Case Rep. (2025) 19:503. doi: 10.1186/s13256-025-05599-z 41084057 PMC12519873

[5] Berger MH Haidar YM . Malignant peripheral nerve-sheath tumor. N Engl J Med. (2021) 385:e23. doi: 10.1056/nejmicm2033082 34387968

[6] Al-Mistarehi AH Zaitoun KJ Khalifeh J Saint-Germain MA Horowitz MA Ghaith AK . An assessment of surgical outcomes in Malignant peripheral nerve sheath tumors: a systematic review and meta-analysis of surgical interventions. Cancers (Basel). (2025) 17. doi: 10.3390/cancers17121997 40563647 PMC12190973

[7] Pemov A Li H Presley W Wallace MR Miller DT . Genetics of human Malignant peripheral nerve sheath tumors. Neuro-Oncol Adv. (2020) 2:i50–61. doi: 10.1093/noajnl/vdz049 32642732 PMC7317054

[8] Sugita S Aoyama T Emori M Kido T Takenami T Sakuraba K . Assessment of H3K27me3 immunohistochemistry and combination of NF1 and p16 deletions by fluorescence in situ hybridization in the differential diagnosis of Malignant peripheral nerve sheath tumor and its histological mimics. Diagn Pathol. (2021) 16:79. doi: 10.1186/s13000-021-01140-0 34461930 PMC8404283

[9] Odhiambo DA Fan S Hirbe AC . UBR5 in tumor biology: exploring mechanisms of immune regulation and possible therapeutic implications in MPNST. Cancers (Basel). (2025) 17. doi: 10.3390/cancers17020161 39857943 PMC11764400

[10] Magallón-Lorenz M Terribas E Ortega-Bertran S Creus-Bachiller E Fernández M Requena G . Deep genomic analysis of Malignant peripheral nerve sheath tumor cell lines challenges current Malignant peripheral nerve sheath tumor diagnosis. iScience. (2023) 26:106096. doi: 10.1016/j.isci.2023.106096 36818284 PMC9929861

[11] Fertitta L . Living with… neurofibromatosis type 1. Rev Prat. (2024) 74:308–10. 38551878

[12] Nakamura K Takashima S Nakayama T Hagihara A Morimoto A Toyoshima M . Percutaneous arterial prostheses of femoro-femoral bypass: using a new method of Modified Seldinger technique. Nihon Igaku Hoshasen Gakkai Zasshi. (1993) 53:970–2. 8371945

[13] Srinivasan A Naidu V Dhivya P . Arterial line placement using modified Seldinger technique: a novel approach. Indian J Crit Care Med. (2023) 27:515–6. doi: 10.5005/jp-journals-10071-24489 37502299 PMC10369313

[14] Neuville A Chibon F Coindre JM . Grading of soft tissue sarcomas: from histological to molecular assessment. Pathology. (2014) 46:113–20. doi: 10.1097/PAT.0000000000000048 24378389

[15] Navarro O Nunez-Santos E Daneman A Faria P Daltro P . Malignant peripheral nerve-sheath tumor arising in a previously irradiated neuroblastoma: report of 2 cases and a review of the literature. Pediatr Radiol. (2000) 30:176–80. doi: 10.1007/s002470050040 10755757

[16] Imazu M Nakamura Y Nakatani H Kaneda H Okamura K Sato O . Cervicothoracic Malignant peripheral nerve sheath tumor in a 12-year-old girl with neurofibromatosis type 1. Eur J Pediatr Surg. (2006) 16:285–7. doi: 10.1055/s-2006-924339 16981098

[17] Dartnell J Pilling J Ferner R Cane P Lang-Lazdunski L . Malignant triton tumor of the brachial plexus invading the left thoracic inlet: a rare differential diagnosis of pancoast tumor. J Thorac Oncol. (2009) 4:135–7. doi: 10.1097/JTO.0b013e31819151ab 19096322

[18] Lin HW Tieu DD Ferrer K Patel U Reilly BK . Cervicothoracic Malignant peripheral nerve sheath tumor. Ear Nose Throat J. (2011) 90:250–1. doi: 10.1177/014556131109000604 21674466

[19] Yan S Sun Y Sun Y Fan Z Phan K Yang Y . Thoracoscopic transclavicular approach for a large thoracic inlet tumor. Ann Thorac Surg. (2014) 98:e91–3. doi: 10.1016/j.athoracsur.2014.06.096 25282248

[20] Samanci Y Togay HS Yakar R Kabukcuoglu F Celik SE . Acute hydrocephalus due to a primary Malignant peripheral nerve sheath tumor of the cervicothoracic junction: a case report and review of the literature. Neurochirurgie. (2017) 63:91–5. doi: 10.1016/j.neuchi.2016.10.006 28502561

[21] Amirian ES Goodman JC New P Scheurer ME . Pediatric and adult Malignant peripheral nerve sheath tumors: an analysis of data from the surveillance, epidemiology, and end results program. J Neuro-Oncol. (2014) 116:609–16. doi: 10.1007/s11060-013-1345-6 24390465

[22] Knight S Knight TE Santiago T Murphy AJ Abdelhafeez AH . Malignant peripheral nerve sheath tumors-a comprehensive review of pathophysiology, diagnosis, and multidisciplinary management. Children (Basel). (2022) 9. doi: 10.3390/children9010038 35053663 PMC8774267

[23] Mattox AK Douville C Silliman N Ptak J Dobbyn L Schaefer J . Detection of Malignant peripheral nerve sheath tumors in patients with neurofibromatosis using aneuploidy and mutation identification in plasma. Elife. (2022) 11. doi: 10.7554/elife.74238 35244537 PMC9094745

[24] Zhang X Guan W Liang J . Malignant peripheral nerve sheath tumor in early childhood: a case report of a diagnostic challenge. Front Oncol. (2025) 15:1609477. doi: 10.3389/fonc.2025.1609477 40630199 PMC12234525

[25] Lu VM Wang S Daniels DJ Spinner RJ Levi AD Niazi TN . The clinical course and role of surgery in pediatric Malignant peripheral nerve sheath tumors: a database study. J Neurosurg Pediatr. (2022) 29:92–9. doi: 10.3171/2021.7.peds21263 34624851

[26] Lim Z Gu TY Tai BC Puhaindran ME . Survival outcomes of Malignant peripheral nerve sheath tumors (MPNSTs) with and without neurofibromatosis type I (NF1): a meta-analysis. World J Surg Oncol. (2024) 22:14. doi: 10.1186/s12957-023-03296-z 38191386 PMC10775467

[27] Sobczuk P Teterycz P Czarnecka AM Świtaj T Koseła-Paterczyk H Kozak K . Malignant peripheral nerve sheath tumors - outcomes and prognostic factors based on the reference center experience. Surg Oncol. (2020) 35:276–84. doi: 10.1016/j.suronc.2020.09.011 32949967

[28] Thway K Fisher C . Malignant peripheral nerve sheath tumor: pathology and genetics. Ann Diagn Pathol. (2014) 18:109–16. doi: 10.1016/j.anndiagpath.2013.10.007 24418643

[29] Coindre JM Terrier P Guillou L Le Doussal V Collin F Ranchère D . Predictive value of grade for metastasis development in the main histologic types of adult soft tissue sarcomas: a study of 1240 patients from the French Federation of Cancer Centers Sarcoma Group. Cancer. (2001) 91:1914–26. doi: 10.1002/1097-0142(20010515)91:10<1914::aid-cncr1214>3.0.co;2-3 11346874

[30] Zou C Smith KD Liu J Lahat G Myers S Wang WL . Clinical, pathological, and molecular variables predictive of Malignant peripheral nerve sheath tumor outcome. Ann Surg. (2009) 249:1014–22. doi: 10.1097/sla.0b013e3181a77e9a 19474676

[31] Karamchandani JR Nielsen TO van de Rijn M West RB . Sox10 and S100 in the diagnosis of soft-tissue neoplasms. Appl Immunohistochem Mol Morphol. (2012) 20:445–50. doi: 10.1097/pai.0b013e318244ff4b 22495377

[32] Miettinen MM Antonescu CR Fletcher C Kim A Lazar AJ Quezado MM . Histopathologic evaluation of atypical neurofibromatous tumors and their transformation into Malignant peripheral nerve sheath tumor in patients with neurofibromatosis 1-a consensus overview. Hum Pathol. (2017) 67:1–10. doi: 10.1017/9781107707504.025 28551330 PMC5628119

[33] Weiss SW Nickoloff BJ . CD-34 is expressed by a distinctive cell population in peripheral nerve, nerve sheath tumors, and related lesions. Am J Surg Pathol. (1993) 17:1039–45. doi: 10.1097/00000478-199310000-00009 7690524

[34] Watanabe T Oda Y Tamiya S Masuda K Tsuneyoshi M . Malignant peripheral nerve sheath tumour arising within neurofibroma. An immunohistochemical analysis in the comparison between benign and Malignant components. J Clin Pathol. (2001) 54:631–6. doi: 10.1136/jcp.54.8.631 11477120 PMC1731495

[35] Shimada S Tsuzuki T Kuroda M Nagasaka T Hara K Takahashi E . Nestin expression as a new marker in Malignant peripheral nerve sheath tumors. Pathol Int. (2007) 57:60–7. doi: 10.1111/j.1440-1827.2006.02059.x 17300669

[36] Cui Q Zhang F Liu J Xu J Wu H Xu F . Genomic profiling and pathological assessment of Malignant peripheral nerve sheath tumors. J Cancer Res Clin Oncol. (2025) 151:165. doi: 10.1007/s00432-025-06209-7 40369172 PMC12078426

[37] Verdijk RM den Bakker MA Dubbink HJ Hop WC Dinjens WN Kros JM . TP53 mutation analysis of Malignant peripheral nerve sheath tumors. J Neuropathol Exp Neurol. (2010) 69:16–26. doi: 10.1097/NEN.0b013e3181c55d55 20010306

[38] Pekmezci M Reuss DE Hirbe AC Dahiya S Gutmann DH von Deimling A . Morphologic and immunohistochemical features of Malignant peripheral nerve sheath tumors and cellular schwannomas. Mod Pathol. (2015) 28:187–200. doi: 10.1038/modpathol.2014.109 25189642 PMC6816504

[39] Zhou H Coffin CM Perkins SL Tripp SR Liew M Viskochil DH . Malignant peripheral nerve sheath tumor: a comparison of grade, immunophenotype, and cell cycle/growth activation marker expression in sporadic and neurofibromatosis 1-related lesions. Am J Surg Pathol. (2003) 27:1337–45. doi: 10.1097/00000478-200310000-00006 14508395

[40] Cleven AH Sannaa GA Briaire-de Bruijn I Ingram DR van de Rijn M Rubin BP . Loss of H3K27 tri-methylation is a diagnostic marker for Malignant peripheral nerve sheath tumors and an indicator for an inferior survival. Mod Pathol. (2016) 29:582–90. doi: 10.1038/modpathol.2016.45 26990975 PMC4948583

[41] Prieto-Granada CN Wiesner T Messina JL Jungbluth AA Chi P Antonescu CR . Loss of H3K27me3 expression is a highly sensitive marker for sporadic and radiation-induced MPNST. Am J Surg Pathol. (2016) 40:479–91. doi: 10.1097/pas.0000000000000564 26645727 PMC4882106

[42] Suppiah S Mansouri S Mamatjan Y Liu JC Bhunia MM Patil V . Multiplatform molecular profiling uncovers two subgroups of Malignant peripheral nerve sheath tumors with distinct therapeutic vulnerabilities. Nat Commun. (2023) 14:2696. doi: 10.1038/s41467-023-38432-6 37164978 PMC10172395

[43] Röhrich M Koelsche C Schrimpf D Capper D Sahm F Kratz A . Methylation-based classification of benign and Malignant peripheral nerve sheath tumors. Acta Neuropathol. (2016) 131:877–87. doi: 10.1007/s00401-016-1540-6 26857854

[44] Bonis A Busetto A Pezzuto F Pagliarini G Verzeletti V Pezzella M . Surgical management of intrathoracic triton tumors: insights into emerging molecular and epigenetic mechanisms with a case series of three patients. J Mol Pathol. (2025) 6. doi: 10.3390/jmp6020010 30654563

[45] Inoue M Hokkoku K Matsukura K Hatanaka Y Sato K Oichi T . Malignant peripheral nerve sheath tumor presenting with Pancoast syndrome in a patient with neurofibromatosis type 1. Rinsho Shinkeigaku. (2026) 66:28–33. doi: 10.5692/clinicalneurol.cn-002168 41391863

[46] Lubbers K Hiralal KR Dieleman GC Hagenaar DA Dierckx B Legerstee JS . Autism spectrum disorder symptom profiles in fragile X syndrome, Angelman syndrome, tuberous sclerosis complex and neurofibromatosis type 1. J Autism Dev Disord. (2024). doi: 10.1007/s10803-024-06557-2 39395123 PMC12864356

[47] Georgescu EF Stănescu L Georgescu AC Dumitrescu D Foarfă C Călin G . Bone abnormalities occurring in the follow-up of the patients with neurofibromatosis type 1. Rom J Morphol Embryol. (2007) 48:249–56. 17914491

[48] Năstase F Radaschin DS Niculeț E Brădeanu AV Verenca MC Nechita A . Orthopaedic manifestations of neurofibromatosis type 1: a case report. Exp Ther Med. (2022) 23:135. doi: 10.3892/etm.2021.11058 35069816 PMC8756425

[49] Salamon J Widemann BC Gross AM Baldwin A Farschtschi SC Mautner VF . Malignant peripheral nerve sheath tumors in neurofibromatosis type 1 arise from distinct nodular lesions: a retrospective imaging analysis. Neuro-Oncol Adv. (2025) 7:vdaf201. doi: 10.1093/noajnl/vdaf201 41473742 PMC12746603

[50] Matsumine A Kusuzaki K Nakamura T Nakazora S Niimi R Matsubara T . Differentiation between neurofibromas and Malignant peripheral nerve sheath tumors in neurofibromatosis 1 evaluated by MRI. J Cancer Res Clin Oncol. (2009) 135:891–900. doi: 10.1007/s00432-008-0523-y 19101731 PMC12160187

[51] Jansma C Wan X Acem I Spaanderman DJ Visser JJ Hanff D . Preoperative classification of peripheral nerve sheath tumors on MRI using radiomics. Cancers (Basel). (2024) 16. doi: 10.3390/cancers16112039 38893158 PMC11170987

[52] Van Herendael BH Heyman SR Vanhoenacker FM De Temmerman G Bloem JL Parizel PM . The value of magnetic resonance imaging in the differentiation between Malignant peripheral nerve-sheath tumors and non-neurogenic Malignant soft-tissue tumors. Skeletal Radiol. (2006) 35:745–53. doi: 10.1007/s00256-006-0160-y 16775712

[53] Yamada N Kato H Kawaguchi M Suzui N Miyazaki T Nagano A . Magnetic resonance imaging features for differentiating low-grade and high-grade Malignant peripheral nerve sheath tumors. J Comput Assist Tomogr. (2024) 48:436–42. doi: 10.1097/rct.0000000000001569 38083833

[54] Matsunaga K Kikuno M Sakamoto H Okada H Hashimoto T Honda S . A cerebral embolism caused by a Malignant peripheral nerve sheath tumor in a patient with neurofibromatosis type 1. Intern Med. (2024) 63:3087–91. doi: 10.2169/internalmedicine.2996-23 38569908 PMC11637803

[55] Chaudhary N Borker A . Metronomic therapy for Malignant peripheral nerve sheath tumor in neurofibromatosis type 1. Pediatr Blood Cancer. (2012) 59:1317–9. doi: 10.1002/pbc.24245 22745048

[56] Furuzono N Togami S Kitazono I Nishikawa T Tanimoto A Kobayashi H . Malignant peripheral nerve sheath tumor of the cervix in an adolescent with neurofibromatosis type 1: a case report and review of literature. J Obstet Gynaecol Res. (2024) 50:2372–6. doi: 10.1111/jog.16139 39497595 PMC11608839

[57] Qian L Ye X Sun J . Retroperitoneal Malignant peripheral nerve sheath tumor in a patient with neurofibromatosis type 1: a case report. BMC Surg. (2026). doi: 10.1186/s12893-026-03495-x 41526870 PMC12888607

[58] Liu P Sun Y . Surgical management of giant neurofibroma in a pediatric patient: a case report. Sci Prog. (2025) 108:368504251357509. doi: 10.1177/00368504251357509 40620148 PMC12235097

[59] Holtkamp N Malzer E Zietsch J Okuducu AF Mucha J Mawrin C . EGFR and erbB2 in Malignant peripheral nerve sheath tumors and implications for targeted therapy. Neuro Oncol. (2008) 10:946–57. doi: 10.1215/15228517-2008-053 18650488 PMC2719009

[60] Lemberg KM Wang J Pratilas CA . From genes to -omics: the evolving molecular landscape of Malignant peripheral nerve sheath tumor. Genes (Basel). (2020) 11. doi: 10.3390/genes11060691 32599735 PMC7349243

[61] McGivney GR Brockman QR Borcherding N Scherer A Rauckhorst AJ Gutierrez WR . Somatic CRISPR tumorigenesis and multiomic analysis reveal a pentose phosphate pathway disruption vulnerability in MPNSTs. Sci Adv. (2025) 11:eadu2906. doi: 10.1126/sciadv.adu2906 40802750 PMC12346278

[62] Yu Y Wei C Yue M Zhang C Wang Y Wang Z . From benign neurofibromas to Malignant peripheral nerve sheath tumors (MPNST): a gaming among multiple factors. Cell Oncol (Dordr). (2025) 48:841–57. doi: 10.1007/s13402-025-01054-9 40172801 PMC12238183

[63] Fletcher JS Pundavela J Ratner N . After Nf1 loss in Schwann cells, inflammation drives neurofibroma formation. Neuro-Oncol Adv. (2020) 2:i23–32. doi: 10.1093/noajnl/vdz045 32642730 PMC7317060

[64] Demiroz SM Sayan M Celik A . Giant tumors of the posterior mediastinum: a narrative review of surgical treatment. Mediastinum. (2022) 6:36. doi: 10.21037/med-21-39 36582978 PMC9792830

[65] Farma JM Porpiglia AS Vo ET . Benign neurogenic tumors. Surg Clin North Am. (2022) 102:679–93. doi: 10.1016/j.suc.2022.04.007 35952696

[66] Lecompte JF Sarnacki S Irtan S Piolat C Scalabre A Talon I . Thoracoscopy for pediatric thoracic neurogenic tumors-a European multi-center study. Cancers (Basel). (2023) 15. doi: 10.3390/cancers15225467 38001727 PMC10670815

[67] Niedermaier B Griffo R Grott M Deissner H Muley T Neumann JO . Robotic thoracic surgery for neurogenic tumors. J Neurosurg. (2024) 141:1369–77. doi: 10.3171/2024.3.JNS232860 38820608

[68] Grunenwald D Spaggiari L Girard P Baldeyrou P . Transmanubrial approach to the thoracic inlet. J Thorac Cardiovasc Surg. (1997) 113:958–9. doi: 10.1016/S0022-5223(97)70276-9 9159636

[69] Nemoto Y Kuroda K Mori M Kanayama M Kuwata T Takenaka M . Robot-assisted thoracoscopic resection of a posterior mediastinal tumor with preserving the artery of Adamkiewicz. Surg Case Rep. (2022) 8:129. doi: 10.1186/s40792-022-01487-6 35790581 PMC9256886

[70] García-López A Iborra A . Transmanubrial transclavicular approach in tumors of the brachial plexus. Ann Plast Surg. (2011) 67:387–90. doi: 10.1097/SAP.0b013e318226b4dc 21750455

[71] Sanker V Gonzalez-Suarez AD Innocenti N Cavagnaro MJ Jeon I Zygourakis C . Patient-specific 3D reconstruction models for sacral tumor resection: illustrative cases. J Neurosurg Case Lessons. (2025) 9. doi: 10.3171/CASE2522 PMC12147658 40489946

[72] Iwai C Nozawa S Fushimi K Yamada K Akiyama H . Surgical management of intraosseous neurofibroma in cervical spine: a case report. JBJS Case Connect. (2024) 14. doi: 10.2106/jbjs.cc.23.00480 38341863

[73] Somaiah N Paudyal B Winkler RE Van Tine BA Hirbe AC . Malignant peripheral nerve sheath tumor, a heterogeneous, aggressive cancer with diverse biomarkers and no targeted standard of care: review of the literature and ongoing investigational agents. Target Oncol. (2024) 19:665–78. doi: 10.1007/s11523-024-01078-5 38954182 PMC11392982

[74] Watson KL Al Sannaa GA Kivlin CM Ingram DR Landers SM Roland CL . Patterns of recurrence and survival in sporadic, neurofibromatosis type 1-associated, and radiation-associated Malignant peripheral nerve sheath tumors. J Neurosurg. (2017) 126:319–29. doi: 10.3171/2015.12.jns152443 27035165 PMC5045773

[75] Kroep JR Ouali M Gelderblom H Le Cesne A Dekker T Van Glabbeke M . First-line chemotherapy for Malignant peripheral nerve sheath tumor (MPNST) versus other histological soft tissue sarcoma subtypes and as a prognostic factor for MPNST: an EORTC soft tissue and bone sarcoma group study. Ann Oncol. (2011) 22:207–14. doi: 10.1093/annonc/mdq338 20656792 PMC3003614

[76] Zehou O Fabre E Zelek L Sbidian E Ortonne N Banu E . Chemotherapy for the treatment of Malignant peripheral nerve sheath tumors in neurofibromatosis 1: a 10-year institutional review. Orphanet J Rare Dis. (2013) 8:127. doi: 10.1186/1750-1172-8-127 23972085 PMC3766199

[77] Frustaci S Gherlinzoni F De Paoli A Bonetti M Azzarelli A Comandone A . Adjuvant chemotherapy for adult soft tissue sarcomas of the extremities and girdles: results of the Italian randomized cooperative trial. J Clin Oncol. (2001) 19:1238–47. doi: 10.1200/jco.2001.19.5.1238 11230464

[78] Ferner RE Gutmann DH . International consensus statement on Malignant peripheral nerve sheath tumors in neurofibromatosis. Cancer Res. (2002) 62:1573–7. 11894862

[79] Kahn J Gillespie A Tsokos M Ondos J Dombi E Camphausen K . Radiation therapy in management of sporadic and neurofibromatosis type 1-associated Malignant peripheral nerve sheath tumors. Front Oncol. (2014) 4:324. doi: 10.3389/fonc.2014.00324 25452937 PMC4233912

[80] Wang D Zhang Q Eisenberg BL Kane JM Li XA Lucas D . Significant reduction of late toxicities in patients with extremity sarcoma treated with image-guided radiation therapy to a reduced target volume: results of radiation therapy oncology group RTOG-0630 trial. J Clin Oncol. (2015) 33:2231–8. doi: 10.1200/jco.2014.58.5828 25667281 PMC4486342

[81] Wong WW Hirose T Scheithauer BW Schild SE Gunderson LL . Malignant peripheral nerve sheath tumor: analysis of treatment outcome. Int J Radiat Oncol Biol Phys. (1998) 42:351–60. doi: 10.1016/s0360-3016(98)00223-5 9788415

[82] Pisters PW Harrison LB Leung DH Woodruff JM Casper ES Brennan MF . Long-term results of a prospective randomized trial of adjuvant brachytherapy in soft tissue sarcoma. J Clin Oncol. (1996) 14:859–68. doi: 10.1007/0-387-22744-x_55 8622034

