# Supplementary material for: Giant cervicothoracic malignant peripheral nerve sheath tumor in neurofibromatosis type 1: a case report of multidisciplinary management and literature review
Source: Front Oncol. 2026 Jul 10;16:1883580. doi: 10.3389/fonc.2026.1883580 (PMC13397498; doi:10.3389/fonc.2026.1883580)
Supplement: Supplementary file 1 [file DataSheet1.pdf]

Supplementary Material

1 Supplementary Figures and Tables

1.1 Figure 1 Caption: 12-month contrast-enhanced MRI and CT outcomes

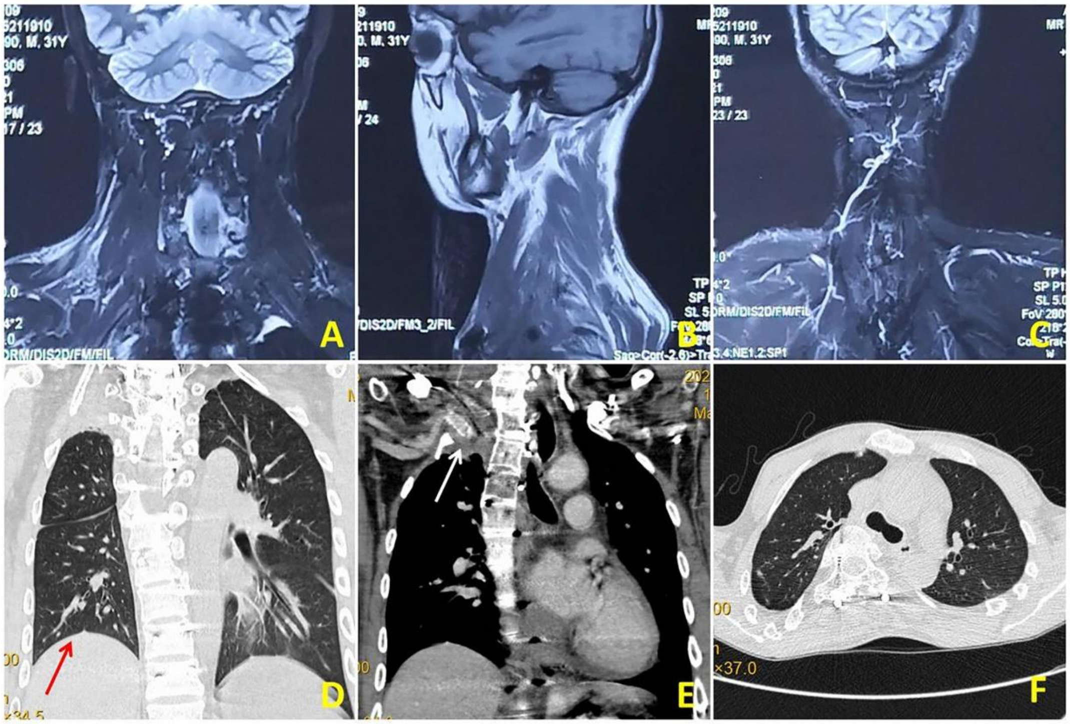

Figure legend: Contrast-enhanced MRI (A-C) and CT outcomes (D-F) at 12-month follow-up revealed no tumor recurrence or pulmonary metastasis. Contrast-enhanced CT demonstrated a mild hemidiaphragm elevation (red arrow), as well as the patent right subclavian artery graft (white arrow) without stenosis or thrombosis,

1.2 Figure 2 Caption: PRISMA-style workflow of the search methodology

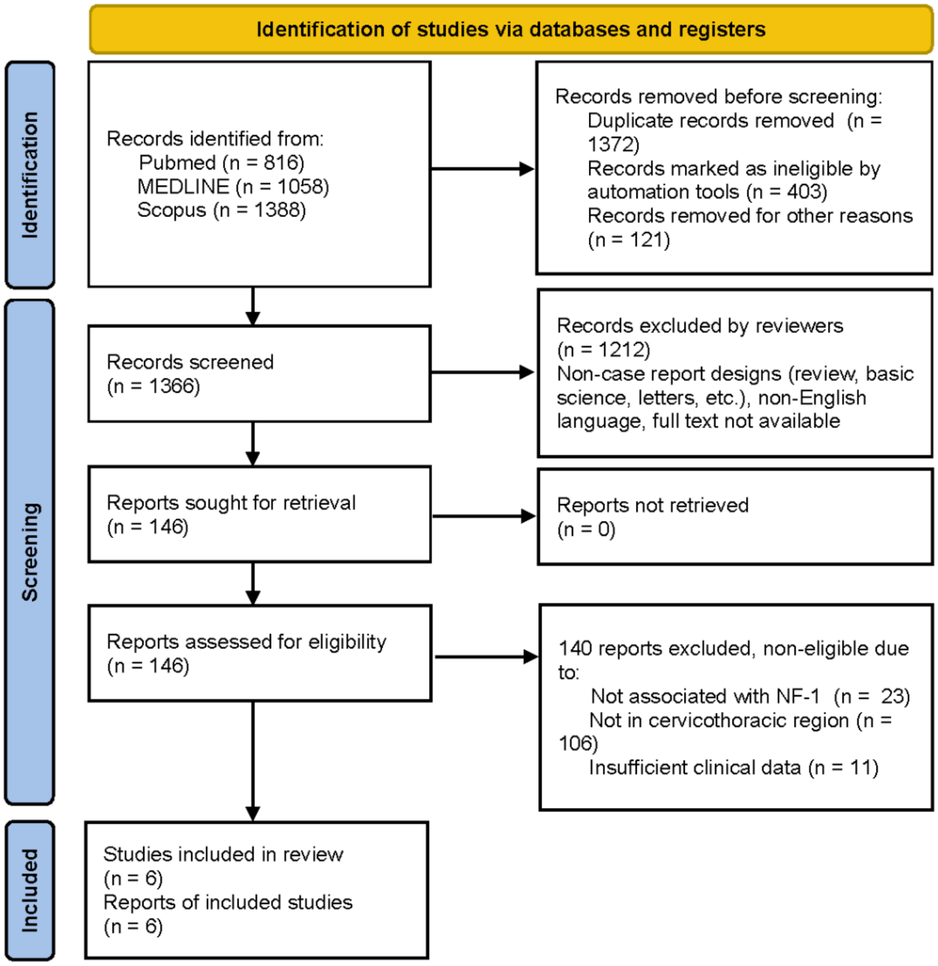

Figure legend: A total of 3262 records were initially identified and 146 full-text articles were retrieved and assessed for eligibility. 6 articles reporting cervicothoracic MPNSTs were further analyzed.
